# A Multiple Compression Approach using Attribute-based Signatures

**DOI:** 10.12688/openreseurope.19247.1

**Published:** 2025-02-10

**Authors:** Constantinos Costa, Panos Chrysanthis, Herodotos Herodotou, Marios Costa, Efstathios Stavrakis, Nicolas Nicolaou

**Affiliations:** 1 Rinnoco Ltd, Limassol, 3047, Cyprus; 2 Electrical Engineering and Computer Engineering and Informatics, Cyprus University of Technology, Limassol, 3036, Cyprus; 3 Algolysis Ltd, Limassol, 4630, Cyprus

**Keywords:** big data, signature based, compression, column stores, hybrid store

## Abstract

**Background:**

With the increasing volume of data collected for advanced analytical and AI applications, data storage remains a significant challenge. Despite advancements in storage technologies, the cost of maintaining vast datasets continues to grow. Compression techniques have been widely used to address this issue, but existing systems primarily rely on a single, typically lossless method, which limits adaptability to varying data characteristics.

**Methods:**

This paper introduces COMPASS, a multiple compression approach that applies different compression techniques to different subsets of data within a database. COMPASS partitions relational data into rows or columns and selects the most suitable compression scheme for individual columns or column groups. Two versions of COMPASS are proposed:

(i) COMPASS-D, which utilizes K-Means clustering based on data values; and (ii) COMPASS-E, which employs K-Means clustering based on column entropy to group similar columns efficiently.

The effectiveness of COMPASS is evaluated using the Envmon dataset, a real-world environmental monitoring database, and compared against monolithic compression methods.

**Results:**

Experimental results demonstrate that COMPASS significantly reduces disk space usage compared to traditional compression techniques. COMPASS-E achieves superior performance in terms of compression time and proximity to the optimal compression ratio, outperforming COMPASS-D. In worst-case scenarios, COMPASS methods offer 22% more savings compared to baseline techniques, with best-case savings reaching 56% (~2× improvement).

**Conclusion:**

The proposed COMPASS framework offers a flexible and adaptive approach to database compression by leveraging multiple schemes tailored to different data subsets. This results in improved storage efficiency and reduced computational overhead. Future work will explore additional data characteristics and clustering methods to further enhance COMPASS’s adaptability and efficiency.

## 1 Introduction

In the current era of AI that transforms the way we live and work, data management and processing techniques are continuously challenged as AI methods effectiveness depends on the availability of vast amounts of data ^1^. Despite the annual doubling of electronically stored data volume, the cost of storage capacity decreases at a rate of less than one-fifth per year ^2^. For this reason, both lossless ^3^ and lossy ^4^ compression techniques have been developed and optimized to reduce the data storage for specific applications as well as for general use.

The current approach to database compression, which we refer to as *monolithic*, utilizes a single, typically lossless method to reduce the disk space of a database. These systems can result in significant savings on storage costs, but cannot adapt to data distribution changes. Thus, they cannot select the best encoding/compression scheme for a given dataset ^5, 6^.

This paper proposes a *multiple compression* approach, dubbed *COMPASS*, that uses different compression techniques for different data subsets in a database. It is anchored on the hypothesis of SIBACO that multi-scheme data compression is more effective for complex big data ^7^. Specifically, COMPASS breaks down relational data into rows or columns and applies the most suitable compression scheme to individual columns or groups of columns.

The paper presents and evaluates two versions of COMPASS: COMPASS-D, which exploits K-Means clustering on data, and COMPASS-E, which exploits K-Means of column entropy to group similar columns. Finally, the best compression scheme is selected for each column group. The experimental results using the Envmon database, a real dataset from the Environmental Monitoring Platform ^1^, show that COMPASS significantly reduces disk space usage compared to monolithic methods, where COMPASS-E outperforms COMPASS-D in terms of compression time as well as proximity to the optimal compression ratio.

The remainder of the paper is structured as follows: Section 2 reviews the current state-of-the-art in compression techniques. Section 3 presents methodology of COMPASS and Section 4 presents our experimental results. Section 5 concludes our paper by discussing the next steps of our work.

## 2 Related work

The realm of big data storage has seen various advancements in compression techniques aimed at reducing storage expenses while preserving efficient data retrieval. We focus on *lossless* compression techniques in this work as the majority of data-intensive applications need to be able to support *exact* queries over stored data.

Lossless data compression can be categorized into four main types. *Dictionary-based compression* uses a dictionary to capture frequently appearing values within the data, replacing them with a shorter index code (examples include LZ77 and LZ78). *Statistical compression* relies on statistical models to estimate the frequency of data values (such as Huffman coding and LZMA). *Transform-based compression* applies mathematical transformations to condense the data into a more compressed format (like Burrows-Wheeler Transform and BZIP2). Finally, *Hybrid compression* merges techniques from the aforementioned categories to enhance compression efficiency (for instance, DEFLATE, which combines LZ77 and Huffman coding).

Big companies have developed specialized lossless compression techniques tailored to their specific application needs. Google’s Snappy ^2^ for example, performs compression through byte-level operations and bit-stream encoding, whereas Facebook’s Zstandard ^3^ employs dictionary-based methods designed for real-time data compression. Additionally, LZ4 ^4^ is recognized for its speed, employing a byte-oriented variant of LZ77. In our experiments, we utilized several well-known compression algorithms from the *zipfile* library, including LZMA, BZIP2, and DEFLATE representing the three compression types mentioned previously.

The research topic of database compression has been under exploration for more than three decades. To meet the substantial demands of OLAP systems processing big data, columnar storage formats have been developed that incorporate compression techniques to reduce storage costs ^8– 10^. Recent advancements in hardware, such as CPUs, RAM, and storage devices, have led to new optimizations that minimize memory accesses by utilizing compression technologies ^11, 12^. Building a compression-aware database management systems can reduce the storage cost and improve the query response time by improving compression ratios ^13^. Additionally, cutting-edge approaches are employing machine learning to manage and store vast amounts of data more efficiently ^14– 17^.

Moreover, leveraging data organization and data types has proven effective in enhancing query speeds and reducing storage requirements through compression ^5, 6, 18^. While applying a uniform compression method across different data partitions can increase compression effectiveness ^19^, we suggest employing a variety of compression strategies to achieve superior results. Although our approach is closer to a black-box technique, it aligns with principles of white-box compression by making the compression mechanisms visible to applications via database metadata ^20^.

The evolving landscape of AI models necessitates continuous storage of large-scale data to iteratively enhance the model accuracy and precision. Storing data indefinitely is a problem that traditional methods (e.g. compression) are addressing by exploiting the computational resources. For instance, while lossless and lossy compression techniques exist, they fall short in supporting big data analytics ^21^. data reduction methods such as sampling ^22^, aggregation for OLAP ^23^, dimensionality reduction techniques like LDA and PCA ^24^, and synopsis/sketches ^25^ provide critical insights by simplifying the complexity of large datasets. Another way to deal with ever-increasing huge amounts of data is to utilize the concept of *data rotting* or *data amnesia* ^26, 27^ to progressively remove detail in stored information as the data ages, thus decreasing the storage cost.

Recent advancements in data compression and querying focuses cloud and big data system, such as BtrBlocks ^21^, optimize decompression and compression ratios for data lakes, enhancing cloud interoperability. Gorilla ^28^, effectively manages vast measurements through float data compression techniques, reducing storage demands significantly. Additionally, Decomposed Bounded Floats ^29^, handles low-precision float data using a decomposed columnar storage format. Moreover, Chimp ^30^ enhances floating point time series data compression, significantly improving compression ratios and access times. These developments underscore the evolving landscape in data compression and querying, providing a crucial context for our COMPASS project.

## 3 Methods

The hypothesis of COMPASS is that multiple data compression scheme can be more effective for complex big data because it allows for incremental compression and partial decompression. This approach utilizes several compression schemes, each tailored to optimize the compression of different data subsets according to their unique characteristics.

COMPASS breaks down relational data into columns and applies the most suitable compression scheme to individual columns or groups of columns. COMPASS uses K-Means clustering to group *similar* columns together, based on their data values or entropy, and then applies the best compression technique to each individual cluster. COMPASS determinesthe compression scheme for a single column or a group of columns by exhaustively testing all possible combinations.

**Figure 1.**
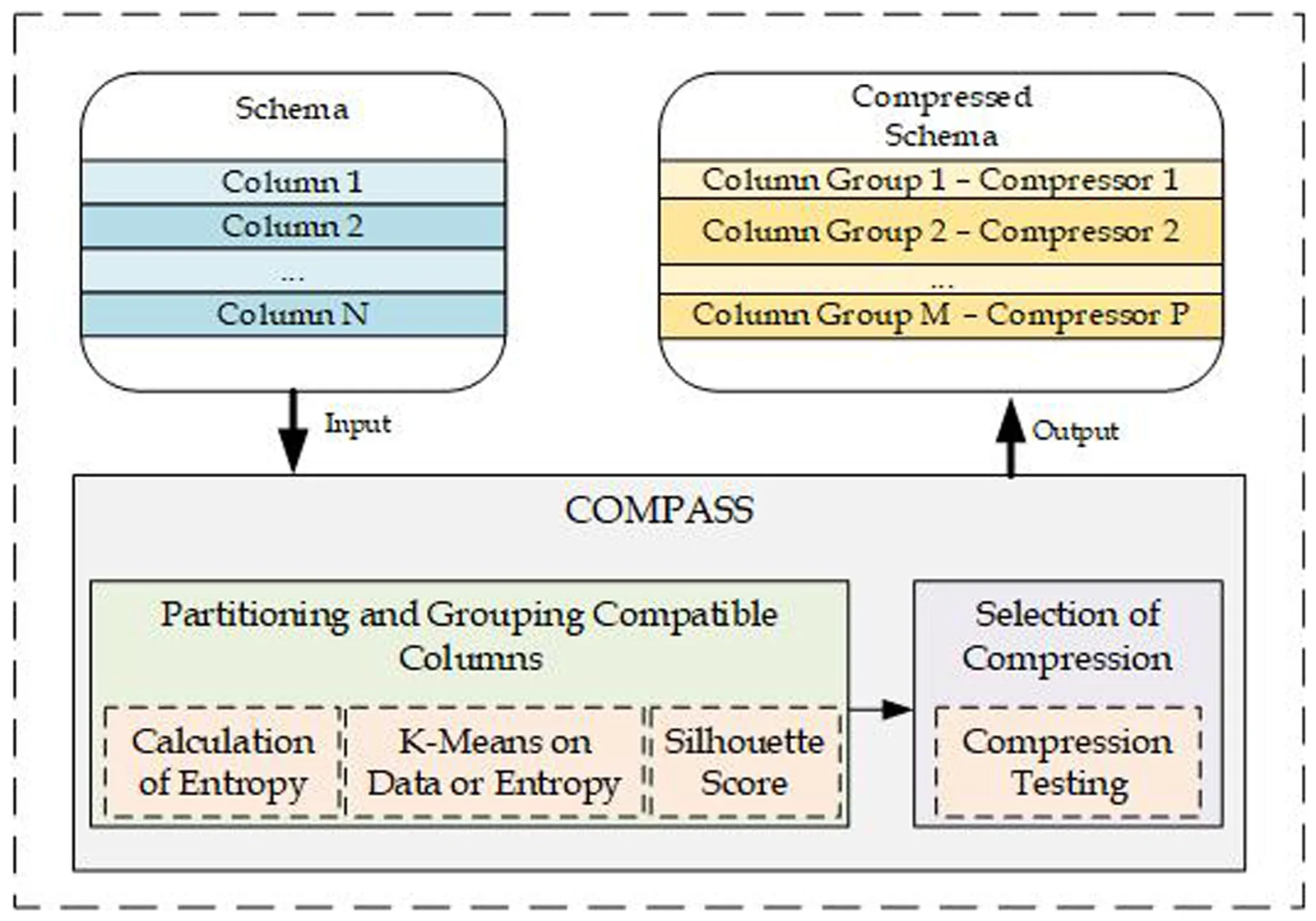
COMPASS operates in two phases: (i) partitioning and grouping; and (ii) choosing the most effective compression method.

Next, we elaborate on COMPASS’ two phases, illustrated in Figure 1: (i) partitioning and grouping compatible columns; and (ii) selection of best performing compression.

### 3.1 Segmentation and clustering of columns

In the first stage, COMPASS partitions each table in the database into a set of columns and utilizes K-Means as the basic method to create groups of similar columns. We apply K-Means in two ways that lead to two different versions of COMPASS: *COMPASS-D*, which utilizes K-Means clustering on data values, and *COMPASS-E*, which exploits K-Means clustering on column entropy to group similar columns. The latter is very fast as it reduces dimensionality through entropy. Specifically, *COMPASS-E* uses Shannon’s entropy ^31^ to identify the compressibility of the columns.

To verify and evaluate our proposed approach, we use the *silhouette coefficient* ^32^ to select the number of clusters for the K-Means algorithm. Particularly, the silhouette coefficient measures the quality of the clusters based on the distance within the cluster and the average distance to the nearest cluster for each data point. The silhouette coefficient for a data point is calculated using the following formula:

$$S_{i}=\frac{y_{i}-x_{i}}{max\{x_{i},y_{i}\}}$$

where:

- *x _i_* is the average distance between the data point *i* and the rest of points within the cluster.
- *y _i_* is the minimum average distance from the data point *i* to points in a different cluster.

### 3.2 Selection of best performing compression

In the second stage, COMPASS exhaustively searches for the most suitable compression scheme for the combinations of clusters with low silhouette scores and all compression schemes (e.g., DEFLATED, BZIP2, LZMA).

## 4 Results & discussion

This section discusses the results and provides details about the experimental setup, including datasets and techniques used for the experimental evaluation.

### 4.1 Experimental methodology

In our experimentation, we chose the same readily available compression algorithms (i.e., LZMA, BZIP2, and DEFLATE) from the *zipfile* library, used in SIBACO.

We utilized K-Means to group the columns, after scaling the input data using the StandardScaler, and encoding all string columns using the OrdinalEncoder from the *sklearn 1.4.2* library.

**Compared Techniques:** Our goal in the first experimental series is the comparison of the following four techniques:

*BASELINE*: This baseline technique compresses the data without considering the data characteristics, using the best compression scheme from the *zipfile* library for a given table.

*SIBACO*: This is our previous technique that employs multiple compression schemes ^7^. SIBACO splits the columns into only two groups based on their entropy and applies the best compression scheme to each column group.

*COMPASS-D*: This is our first proposed technique, which uses KMeans clustering to group of columns based on data values to achieve the best possible compression ratio using multiple schemes if needed.

*COMPASS-E*: This is our second proposed technique that applies K-Means clustering based on the entropy of the columns, which has significantly lower computation complexity than COMPASS-D.

### 4.2 Experimental testbed

To validate our proposed ideas and evaluate COMPASS, we conduct the following experiments over two Ubuntu 22.04 server, each featuring 24GB of RAM with Intel(R) Xeon(R) E5-2630 CPU.

**Envmon Dataset:** This is a real dataset collected from the Environmental Monitoring Platform, developed through the STEAM project (Sea Traffic Management in the Eastern Mediterranean) ^5^. The database contains primarily environmental, meteorological and oceanographic data. The dataset was collected over the course of three years and has a total size of ∼1GB.

### 4.3 Experimental results

**Figure 2.**
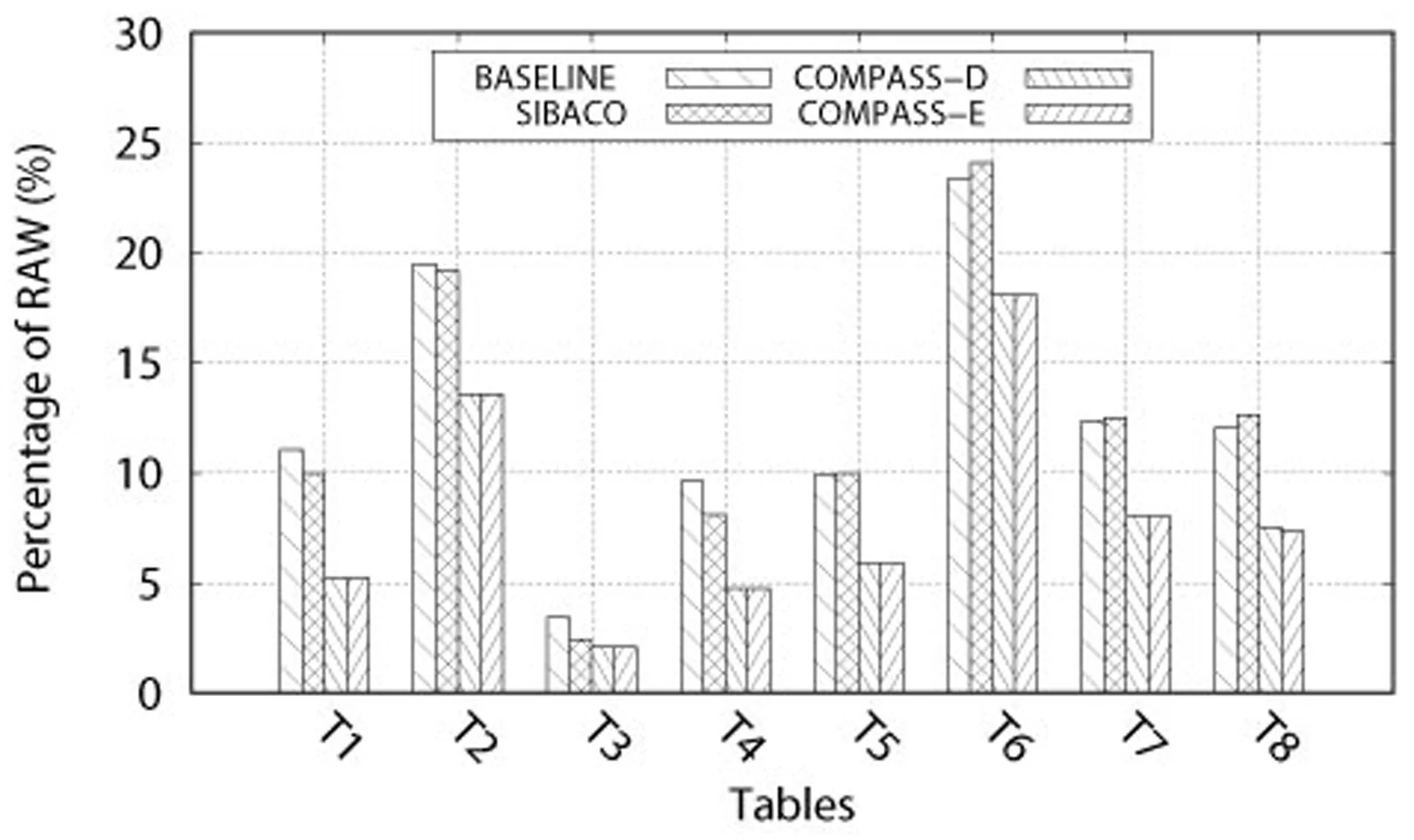
Disk Space for the eight largest tables in the Envmon Database.

Across all tables, COMPASS-D and COMPASS-E consistently demonstrate the most efficient disk space reduction, resulting to 2–18.2% of the original RAW size. In contrast, BASELINE and SIBACO methods reduce the disk space requirements, 4.6–23.4% and 4–23.6% of the original RAW size, respectively. COMPASS-D and COMPASS-E outperform BASELINE and SIBACO by more than 22% in the worst case and 56% (i.e., ∼2×) in the best case, as shown in Figure 2.

### 4.4 Selecting the number of clusters

In this experimental series, we examine the silhouette coefficient while varying the number of clusters in order to verify that the selection of the number of clusters with low silhouette score can yield good compression performance.

**Figure 3.**
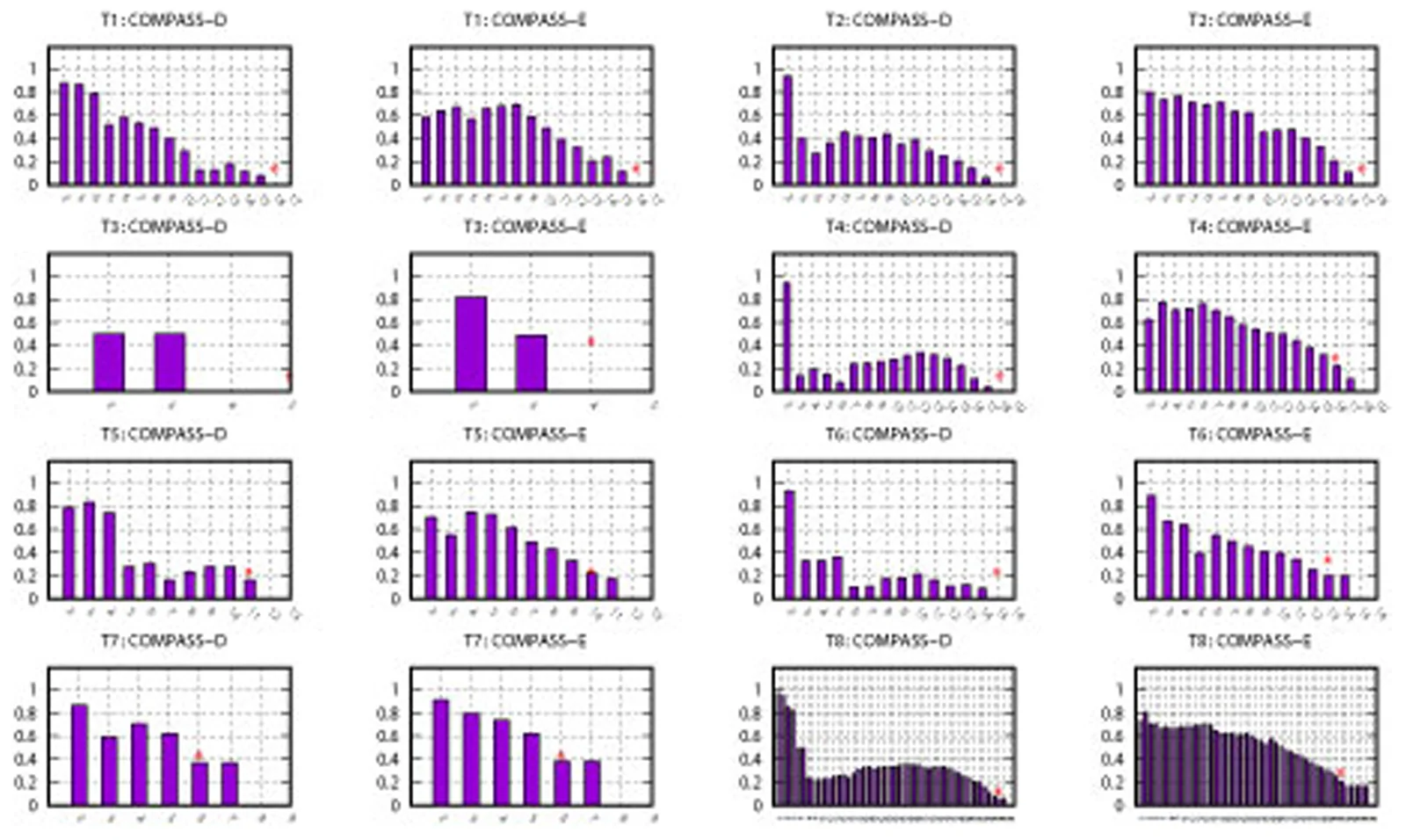
Average Silhouette Score for the eight largest tables in the Envmon Database. The asterisk on top of a bar shows which number of clusters was selected with the best disk space efficiency (X-axis: Number of clusters, Y-axis: Average Silhouette Score).

 Particularly, Figure 3 shows the silhouette coefficient score for the eight largest table in the Envmon Database for all possible numbers of clusters. The asterisk on top of a bar indicates the number of clusters that yields the best compression ratio after the second stage of COMPASS (i.e., Selection of Compression). The clustering with the lowest silhouette coefficient often coincides or is very close to the one with the star, showing that the proposed approach is a very good proxy for finding the (near) optimal compression clustering. It is important to note that the silhouette coefficient is only defined if the number of clusters is between two and N-1, where N is the total number of columns (i.e., the maximum number of clusters).

To better understand the results, we calculate the relative difference between the disk storage of the compressed data for the number of clusters based on the lowest silhouette score and the COMPASS Compression Testing (i.e., the best performing one), using the following formula:

$$\text{Relative}\;\text{Difference}=\frac{\left|D_{S}-D_{C}\right|}{\frac{D_{S}+D_{C}}{2}}\times 100\%$$

where:

- *D _S_* is the disk space for the number of clusters based on the lowest silhouette score.
- *D _C_* is the disk space for the number of clusters based on COMPASS testing.

The maximum relative difference for COMPASS-D is ∼8%, with an average of ∼1%. COMPASS-E exhibits less than ∼1% maximum relative difference and ∼0.1% on average, revealing that COMPASS-E’s results are closer to the optimal compression ratios compared to COMPASS-D.

## 5 Conclusion

This paper presents a novel multiple compression approach, named COMPASS, that uses different compression techniques for different data subsets in a database. We introduce and evaluate two versions of COMPASS, namely COMPASS-D and COMPASS-E, designed to enhance the compression of relational data. COMPASS-D is leveraging K-Means clustering on the data values while COMAPASS-E on the column entropy, to achieve more efficient compression ratios in relational databases. In our experimental evaluation, we observe that COMPASS offers substantial reductions in disk space utilization compared to traditional monolithic methods and our previous work, SIBACO. Specifically, COMPASS-D and COMPASS-E outperform the BASELINE and SIBACO techniques in terms of disk storage savings by more than 22% in the worst case and 56% (i.e., ∼2×) in the best case.

In the future, in addition to entropy and the silhouette coefficient, we plan to explore other data characteristics and indicators to enhance the speed and efficiency of COMPASS. Additionally, we aim to use these metrics to construct a comprehensive knowledge base, providing deeper insights into attribute-based compression signatures.

## Ethics and consent

Ethical approval and consent were not required.

## Data Availability

The data supporting the findings of this study have been deposited in Zenodo and are publicly available at https://doi.org/10.5281/zenodo.14751777 ^33^. Data are available under the terms of the Creative Commons Attribution 4.0 International license (CC-BY 4.0).

## Software availability

- Source code available from: https://github.com/Rinnoco/compass_com
- Archived software available from: https://doi.org/10.5281/zenodo.14759015
- License: Apache 2.0

The software used in this study has been archived in Zenodo and is publicly available at https://doi.org/10.5281/zenodo.14759015. It is released under the Apache 2.0 license ^34^.
