# A Multiple Compression Approach using Attribute-based Signatures

**DOI:** 10.12688/openreseurope.19247.2

**Published:** 2026-09-08

**Authors:** Constantinos Costa, Panos Chrysanthis, Herodotos Herodotou, Marios Costa, Efstathios Stavrakis, Nicolas Nicolaou

**Affiliations:** 1 Rinnoco Ltd, Limassol, 3047, Cyprus; 2 Electrical Engineering and Computer Engineering and Informatics, Cyprus University of Technology, Limassol, 3036, Cyprus; 3 Algolysis Ltd, Limassol, 4630, Cyprus

**Keywords:** big data, signature based, compression, column stores, hybrid store

## Abstract

**Background:**

With the increasing volume of data collected for advanced analytical and AI applications, data storage remains a significant challenge. Despite advancements in storage technologies, the cost of maintaining vast datasets continues to grow. Compression techniques have been widely used to address this issue, but existing systems primarily rely on a single, typically lossless method, which limits adaptability to varying data characteristics.

**Methods:**

This paper introduces COMPASS, a multiple compression approach that applies different compression techniques to distinct subsets of data within a database. COMPASS partitions relational data into rows or columns and selects the most suitable compression scheme for each column or column group. Two versions of COMPASS are proposed: (i) COMPASS-D, which utilizes K-Means clustering based on data values; and (ii) COMPASS-E, which employs K-Means clustering based on column entropy to group similar columns efficiently. The effectiveness of COMPASS is evaluated using the Envmon dataset, a real-world environmental monitoring database, and compared against monolithic compression methods.

**Results:**

Experimental results demonstrate that COMPASS significantly reduces disk space usage compared to traditional compression techniques. COMPASS-E achieves superior performance in terms of compression time and proximity to the optimal compression ratio, outperforming COMPASS-D. In worst-case scenarios, COMPASS methods offer 22% more savings compared to baseline techniques, with best-case savings reaching 56% (~2× improvement).

**Conclusion:**

The proposed COMPASS framework offers a flexible and adaptive approach to database compression by leveraging multiple schemes tailored to different data subsets. This results in improved storage efficiency and reduced computational overhead. Future work will explore additional data characteristics and clustering methods to further enhance COMPASS’s adaptability and efficiency.

## 1. Introduction

In the current era of AI that transforms the way we live and work, data management and processing techniques are continuously challenged as AI methods’ effectiveness depends on the availability of vast amounts of data. ^1^ Despite the annual doubling of electronically stored data volume, the cost of storage capacity decreases at a rate of less than one-fifth per year. ^2^ For this reason, both lossless ^3^ and lossy ^4^ compression techniques have been developed and optimized to reduce data storage for specific applications as well as for general use.

The current approach to database compression, which we refer to as *monolithic*, utilizes a single, typically lossless method to reduce the disk space of a database. These systems can result in significant savings in storage costs, but cannot adapt to data distribution changes. Thus, they cannot select the best encoding/compression scheme for a given dataset. ^5^^,^^6^

This paper proposes a *multiple compression* approach, dubbed *COMPASS*, that uses different compression techniques for distinct data subsets in a database. It is anchored on the hypothesis of SIBACO that multi-scheme data compression is more effective for complex big data. ^7^ Specifically, COMPASS breaks down relational data into rows or columns and applies the most suitable compression scheme to individual columns or groups of columns.

The paper presents and evaluates two versions of COMPASS: COMPASS-D, which exploits K-Means clustering on data, and COMPASS-E, which exploits K-Means on column entropy to group similar columns. Finally, the best compression scheme is selected for each column group. The experimental results using the Envmon database, a real dataset from the Environmental Monitoring Platform [1], show that COMPASS significantly reduces disk space usage compared to monolithic methods, where COMPASS-E outperforms COMPASS-D in terms of compression time as well as proximity to the optimal compression ratio.

The remainder of the paper is structured as follows: Section 2 reviews the current state-of-the-art in compression techniques. Section 3 presents the methodology of COMPASS, and Section 4 presents our experimental results. Section 5 concludes our paper by discussing the next steps of our work.

## 2. Related Work

The realm of big data storage has seen various advancements in compression techniques aimed at reducing storage expenses while preserving efficient data retrieval. We focus on *lossless* compression techniques in this work as the majority of data-intensive applications need to be able to support *exact* queries over stored data.

Lossless data compression can be categorized into four main types. *Dictionary-based compression* uses a dictionary to capture frequently appearing values within the data, replacing them with a shorter index code (examples include LZ77 and LZ78). *Statistical compression* relies on statistical models to estimate the frequency of data values (such as Huffman coding and LZMA). *Transform-based compression* applies mathematical transformations to condense the data into a more compressed format (like Burrows-Wheeler Transform and BZIP2). Finally, *Hybrid compression* merges techniques from the aforementioned categories to enhance compression efficiency (for instance, DEFLATE, which combines LZ77 and Huffman coding). Previous studies have investigated the automated selection of compression algorithms for heterogeneous datasets, including synthesis-based approaches ^8^ and evolutionary strategies. ^9^ In addition, simple machine-learning methods have been proposed for selecting the most appropriate compression algorithm based on extracted features/characteristics (e.g., entropy, data distribution), following a principle similar to SIBACO. ^10^

Big companies have developed specialized lossless compression techniques tailored to their specific application needs. Google’s Snappy [2] for example, performs compression through byte-level operations and bit-stream encoding, whereas Facebook’s Zstandard [3] employs dictionary-based methods designed for real-time data compression. Additionally, LZ4 [4] is recognized for its speed, employing a byte-oriented variant of LZ77. In our experiments, we utilized several well-known compression algorithms from the *zipfile* library, including LZMA, BZIP2, and DEFLATE, representing the three compression types mentioned previously. However, the framework itself is not limited to these compression schemes and can be extended to incorporate additional modern compressors such as Zstandard or LZ4.

The research topic of database compression has been under exploration for more than three decades. To meet the substantial demands of OLAP systems processing big data, columnar storage formats have been developed that incorporate compression techniques to reduce storage costs. ^11^^–^^13^ Recent advancements in hardware, such as CPUs, RAM, and storage devices, have enabled new optimizations that minimize memory accesses by leveraging compression technologies. ^14^^,^^15^ Building a compression-aware database management system can reduce storage costs and improve query response times by increasing compression ratios. ^16^ Additionally, cutting-edge approaches are employing machine learning to manage and store vast amounts of data more efficiently. ^17^^–^^20^

Moreover, leveraging data organization and data types has proven effective in enhancing query speeds and reducing storage requirements through compression. ^5^^,^^6^^,^^21^ While applying a uniform compression method across different data partitions can increase compression effectiveness, ^22^ we suggest employing a variety of compression strategies to achieve superior results. Although our approach is closer to a black-box technique, it aligns with principles of white-box compression by making the compression mechanisms visible to applications via database metadata. ^23^

The evolving landscape of AI models necessitates continuous storage of large-scale data to iteratively improve model accuracy and precision. Storing data indefinitely is a problem that traditional methods (e.g., compression) address by exploiting the computational resources. For instance, while lossless and lossy compression techniques exist, they fall short in supporting big data analytics. ^24^ Data reduction methods such as sampling, ^25^ aggregation for OLAP, ^26^ dimensionality reduction techniques like LDA and PCA, ^27^ and synopsis/sketches ^28^ provide critical insights by simplifying the complexity of large datasets. Another way to deal with ever-increasing huge amounts of data is to utilize the concept of *data rotting* or *data amnesia* ^29^^,^^30^ to progressively remove detail from stored information as the data ages, thereby decreasing storage costs.

Recent advancements in data compression and querying focus cloud and big data systems, such as BtrBlocks, ^24^ optimizing decompression and compression ratios for data lakes, enhancing cloud interoperability. Gorilla ^31^ effectively manages vast amounts of data through float data compression techniques, reducing storage demands significantly. Additionally, Decomposed Bounded Floats ^32^ handles low-precision float data using a decomposed columnar storage format. Moreover, Chimp ^33^ enhances floating-point time series data compression, significantly improving compression ratios and access times. These developments underscore the evolving landscape in data compression and querying, providing a crucial context for our COMPASS project.

## 3. Methods

The underlying hypothesis of COMPASS is that employing multiple data compression schemes can be more effective for complex big data, as it enables the selection of different compression methods for groups of data with similar characteristics. These groups can then be decompressed individually to meet the requirements of specific queries, without decompressing the entire dataset. This approach utilizes several compression schemes, each tailored to optimize the compression of different data subsets according to their unique characteristics.

COMPASS breaks down relational data into columns and applies the most suitable compression scheme to each column or group of columns. COMPASS uses K-Means clustering to group *similar* columns together, based on their data values or entropy, and then applies the best compression technique to each individual cluster. COMPASS determines the compression scheme for a single column or a group of columns by exhaustively testing all possible combinations.

**Figure 1.**
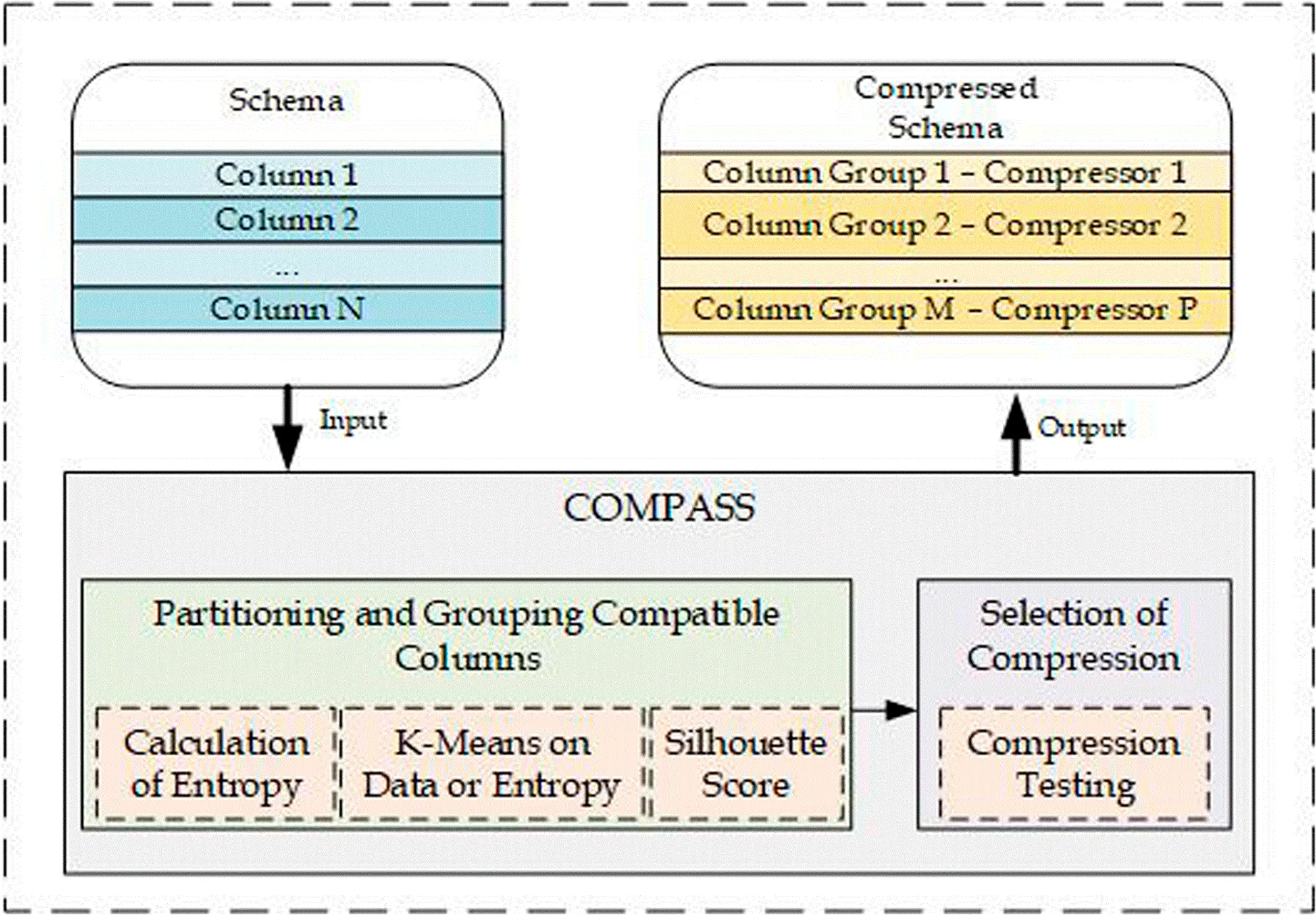
COMPASS operates in two phases: (i) partitioning and grouping; and (ii) choosing the most effective compression method.

Next, we elaborate on COMPASS’ two phases, illustrated in Figure 1: (i) partitioning and grouping compatible columns; and (ii) selection of best performing compression.

### 3.1 Segmentation and clustering of columns

In the first stage, COMPASS partitions each table in the database into a set of columns and utilizes K-Means as the primary method to create groups of similar columns. We apply K-Means in two ways, leading to two different versions of COMPASS: *COMPASS-D *, which utilizes K-Means clustering on data values, and *COMPASS-E *, which exploits K-Means clustering on column entropy to group similar columns. The latter is very fast as it reduces dimensionality through entropy. Specifically, *COMPASS-E* uses Shannon’s entropy ^34^ to identify the compressibility of the columns.

To verify and evaluate our proposed approach, we use the *silhouette coefficient* ^27^ to select the number of clusters for the K-Means algorithm. Particularly, the silhouette coefficient measures the quality of the clusters based on the distance within each cluster and the average distance to the nearest cluster for each data point. The silhouette coefficient for a data point is calculated using the following:

$$S_{i}=\frac{y_{i}-x_{i}}{max\{x_{i},y_{i}\}}$$

where:

• *x _i_* is the average distance between the data point *i* and the rest of the points within the cluster.
• *y _i_* is the minimum average distance from the data point *i* to points in a different cluster.

### 3.2 Selection of best performing compression

In the second stage, COMPASS exhaustively searches for the most suitable compression scheme for the combinations of clusters with low silhouette scores and all compression schemes (e.g., DEFLATED, BZIP2, LZMA). While the silhouette coefficient is typically maximized in clustering tasks, our objective differs. Lower silhouette values indicate clusters that are less strictly separated, which in our context may allow columns with similar compression characteristics to be grouped together even if their statistical profiles differ. Empirically, we observed that such groupings can yield higher compression ratios because the selected compression scheme can exploit shared structural patterns across columns.

## 4. Results & Discussion

This section discusses the results and provides details on the experimental setup, including datasets and techniques used for the experimental evaluation.

### 4.1 Experimental methodology

In our experimentation, we chose the same readily available compression algorithms (i.e., LZMA, BZIP2, and DEFLATE) from the *zipfile* library, used in SIBACO.

We utilized K-Means to group the columns, after scaling the input data with StandardScaler, and encoding all string columns with the OrdinalEncoder from the *sklearn 1.4.2* library. To ensure reproducibility, the K-means clustering algorithm was initialized with a fixed random seed (seed = 42).

**Compared Techniques:** Our goal in the first experimental series is the comparison of the following four techniques:

*BASELINE*: This baseline technique compresses the data without considering the data characteristics, using the best compression scheme from the *zipfile* library for each table.

*SIBACO*: This is our previous technique that employs multiple compression schemes. ^7^ SIBACO splits the columns into only two groups based on their entropy and applies the best compression scheme to each column group.

*COMPASS-D *: This is our first proposed technique, which uses K-Means clustering to group columns based on data values to achieve the best possible compression ratio using multiple schemes if needed.

*COMPASS-E *: This is our second proposed technique, which applies K-Means clustering based on column entropy and has significantly lower computational complexity than COMPASS-D.

### 4.2 Experimental testbed

To validate our proposed ideas and evaluate COMPASS, we conduct the following experiments on two Ubuntu 22.04 servers, each featuring 24GB of RAM and an Intel(R) Xeon(R) E5-2630 CPU.

**Envmon Dataset:** This is a real dataset collected from the Environmental Monitoring Platform, developed under the STEAM project (Sea Traffic Management in the Eastern Mediterranean) [5]. The database contains primarily environmental, meteorological, and oceanographic data. The dataset was collected over the course of three years and has a total size of ∼1GB.

### 4.3 Experimental results

**Figure 2.**
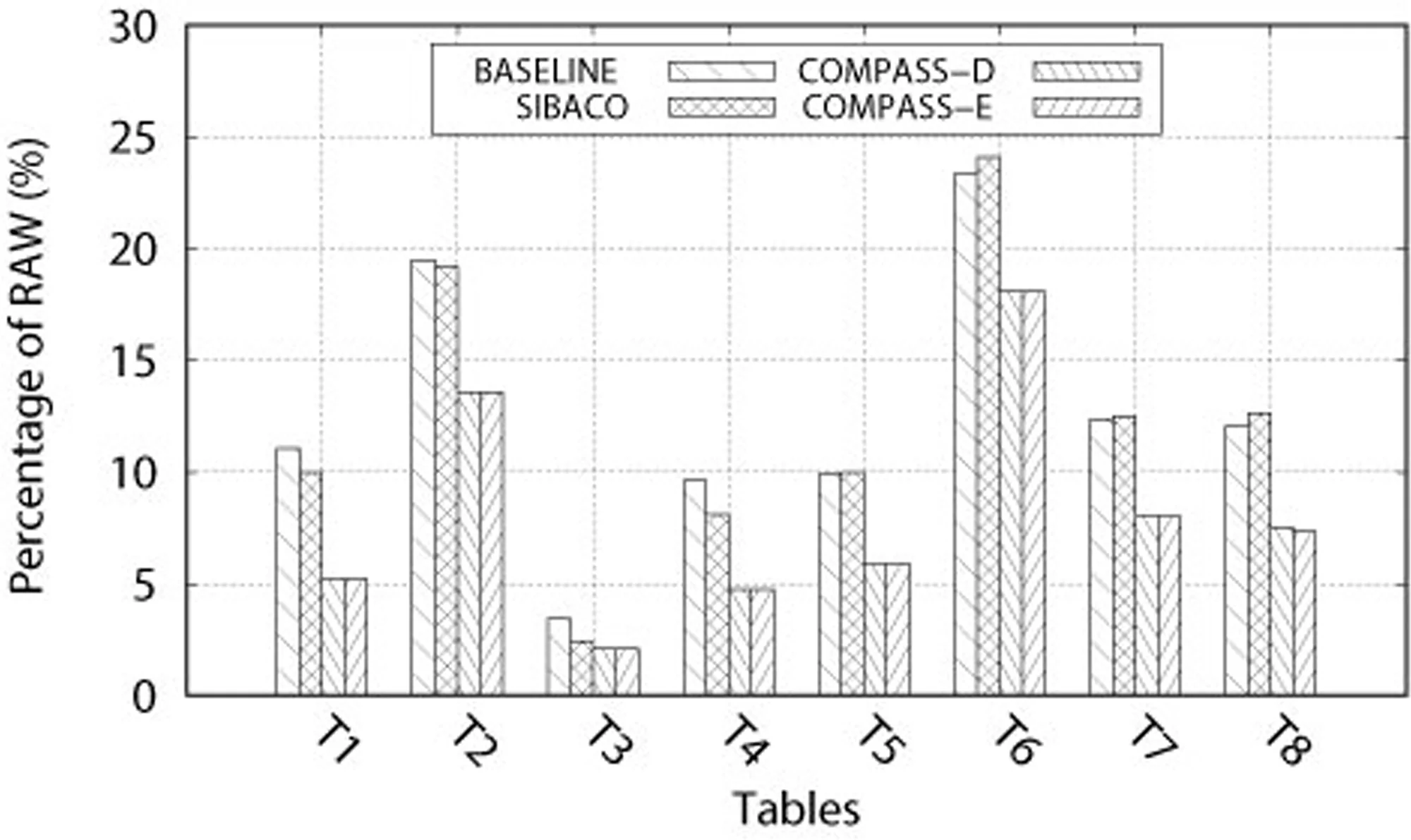
Disk Space for the eight largest tables in the Envmon Database.

Across all tables, COMPASS-D and COMPASS-E consistently achieve the most efficient disk space reduction, resulting in 2–18.2% of the original RAW size. In contrast, the BASELINE and SIBACO methods reduce the disk space requirements by 4.6–23.4% and 4–23.6% of the original RAW size, respectively. COMPASS-D and COMPASS-E outperform BASELINE and SIBACO by more than 22% in the worst case and 56% (i.e., ∼2×) in the best case, as shown in Figure 2.

### 4.4 Selecting the number of clusters

In this experimental series, we examine the silhouette coefficient as we vary the number of clusters to verify that selecting a low-silhouette-score number of clusters can yield good compression performance.

**Figure 3.**
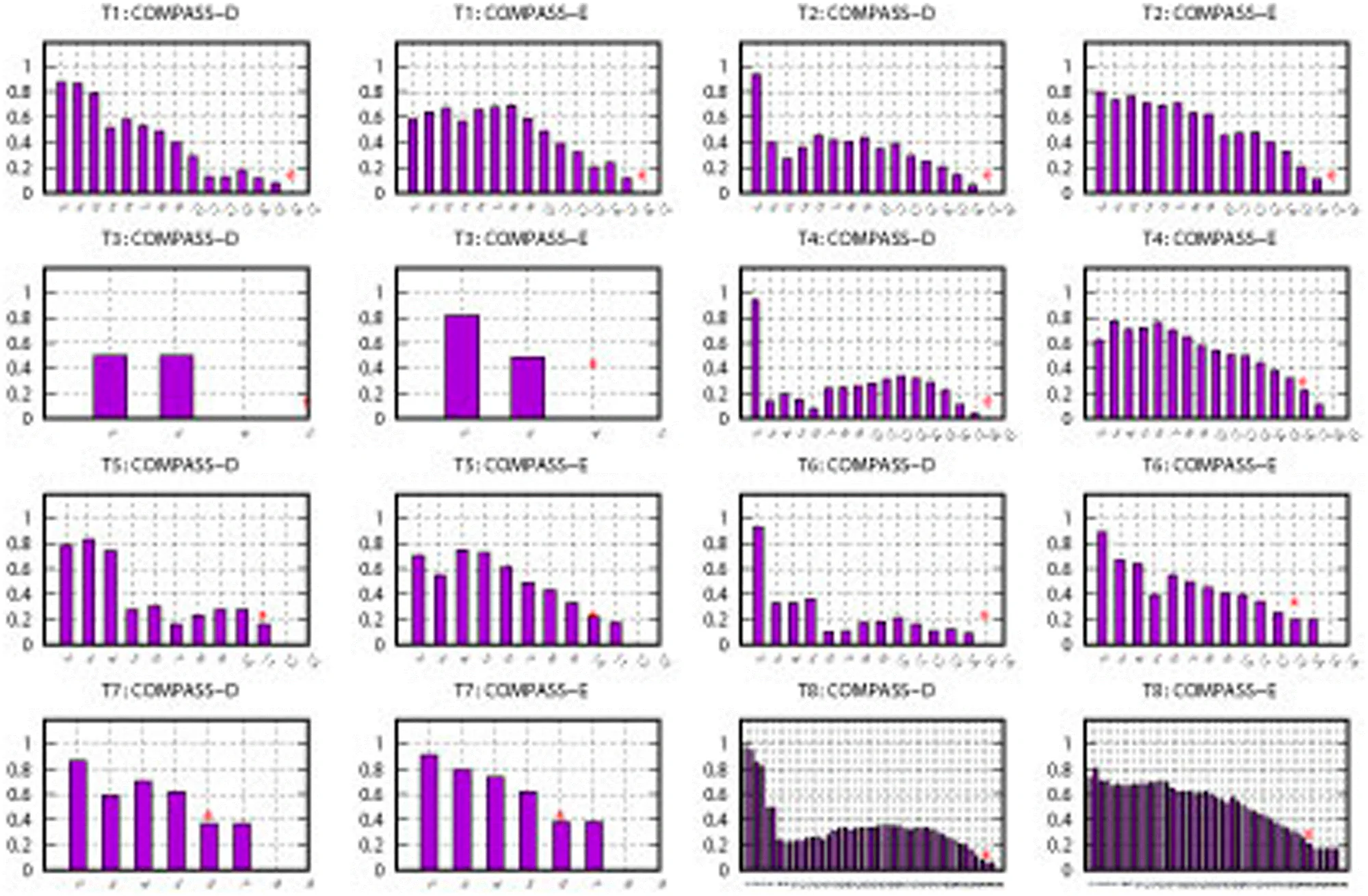
Average Silhouette Score for the eight largest tables in the Envmon Database.

Particularly, Figure 3 shows silhouette coefficient scores for the eight largest tables in the Envmon Database for all possible numbers of clusters. The asterisk on top of a bar indicates the number of clusters that yields the best compression ratio after the second stage of COMPASS (i.e., Selection of Compression). The clustering with the lowest silhouette coefficient often coincides or is very close to the one with the star, showing that the proposed approach is a very good proxy for finding the (near) optimal compression clustering. It is important to note that the silhouette coefficient is only defined if the number of clusters is between two and N-1, where N is the total number of columns (i.e., the maximum number of clusters).

The asterisk on top of a bar shows which number of clusters was selected with the best disk space efficiency (X-axis: Number of clusters, Y-axis: Average Silhouette Score).

To better understand the results, we calculate the relative difference between the disk storage of the compressed data for the number of clusters based on the lowest silhouette score and the COMPASS Compression Testing (i.e., the best performing one), using the following formula:

$$\text{Relative}\;\text{Difference}=\frac{\left|DS-DC\right|}{\frac{DS+DC}{2}}\times 100\%$$

where:

• *D _S_* is the disk space for the number of clusters based on the lowest silhouette score.
• *D _C_* is the disk space for the number of clusters based on COMPASS testing.

The maximum relative difference for COMPASS-D is ∼8%, with an average of ∼1%. COMPASS-E exhibits less than ∼1% maximum relative difference and ∼0.1% on average, revealing that COMPASS-E’s results are closer to the optimal compression ratios compared to COMPASS-D.

### 4.5 Compression and Decompression Time

In addition to the compression ratio, we evaluated the runtime overhead of COMPASS. Table 1 reports compression and decompression times, along with CPU and RAM usage, measured on a machine with 40GB of RAM and a 12th Gen Intel(R) Core i9-12900H processor. Although COMPASS introduces a clustering step, the additional cost remains modest relative to the overall compression process.

**Table 1.** Compression and decompression runtime of the evaluated methods on the Envmon dataset.

Method	Compression Time (s)	Decompression Time (s)	CPU (%)	RAM (MB)
RAW	0.000	0.000	0.000	0.000
Baseline (single scheme)	36.082	0.726	100.805	438.143
COMPASS-D	17.150	0.922	100.640	451.231
COMPASS-E	16.311	0.846	100.645	437.548

## 5. Conclusion

This paper presents a novel multiple compression approach, named COMPASS, that uses different compression techniques for distinct data subsets in a database. We introduce and evaluate two versions of COMPASS, namely COMPASS-D and COMPASS-E, designed to enhance the compression of relational data. COMPASS-D leverages K-Means clustering on the data values, while COMPASS-E uses column entropy to achieve more efficient compression ratios in relational databases. In our experimental evaluation, we observe that COMPASS offers substantial reductions in disk space utilization compared to traditional monolithic methods and our previous work, SIBACO. Specifically, COMPASS-D and COMPASS-E outperform the BASELINE and SIBACO techniques in terms of disk storage savings by more than 22% in the worst case and 56% (i.e., ∼2×) in the best case.

While the results demonstrate the effectiveness of COMPASS on the Envmon dataset, the current evaluation focuses on a single real-world dataset. Although this dataset represents a realistic environmental monitoring workload, additional experiments on datasets with different schema structures and data characteristics would further validate the generality of the approach. Future work will therefore evaluate COMPASS across a broader range of relational datasets and explore integrating additional compression schemes.

Furthermore, in addition to entropy and the silhouette coefficient, we plan to explore other data characteristics and indicators to enhance the speed and efficiency of COMPASS. Additionally, we aim to use these metrics to construct a comprehensive knowledge base that provides deeper insights into attribute-based compression signatures. Lastly, query-time evaluation is an important direction for future work, but it was outside the scope of the current study.

## Ethics and Consent

Ethical approval and consent were not required.

## Data Availability

The data supporting the findings of this study have been deposited in Zenodo and are publicly available at https://doi.org/10.5281/zenodo.14751777. ^35^ Data are available under the terms of the Creative Commons Attribution 4.0 International license (CC-BY 4.0).

## Software Availability

• Source code available from: https://github.com/Rinnoco/compass_com
• Archived software available from: https://doi.org/10.5281/zenodo.14759015
• License: Apache 2.0

The software used in this study has been archived in Zenodo and is publicly available at https://doi.org/10.5281/zenodo.14759015. It is released under the Apache 2.0 license. ^36^

## Acknowledgments

This work is implemented under the programme of social cohesion “THALIA 2021-2027” co-funded by the European Union, through Research and Innovation Foundation, and is also partially supported by the European Union’s Horizon Europe program for Research and Innovation through the HYPER-AI project under Grant No. 101135982. The views, findings, conclusions, or recommendations expressed in this material are solely those of the author(s) and do not necessarily represent those of the sponsors. The funders had no role in study design, data collection and analysis, decision to publish, or preparation of the manuscript.

## Footnotes

^1^
Envmon Database was produced by the STEAM: https://steam.cut.ac.cy

^2^
Snappy: https://google.github.io/snappy/

^3^
Zstandard: https://facebook.github.io/zstd/

^4^
LZ4: https://lz4.github.io/lz4/

^5^
STEAM: https://steam.cut.ac.cy
