# Supplementary material for: A Multiple Compression Approach using Attribute-based Signatures
Source: Open Res Eur. 2026 Sep 8;5:49. Originally published 2025 Feb 10. [Version 2] doi: 10.12688/openreseurope.19247.2 (PMC13571026; doi:10.12688/openreseurope.19247.2)
Supplement: Peer review history

# Reviewer report: Reviewer response for version 2

**DOI:** 10.21956/openreseurope.25401.r80664

**Published:** 2026-09-12

**Authors:** Martin Burtscher

**Affiliations:** 1 Texas State University, San Marcos, Texas, USA

The authors have addressed my previous comments.

Is the study design appropriate and does the work have academic merit?

Yes

Is the work clearly and accurately presented and does it cite the current literature?

Partly

If applicable, is the statistical analysis and its interpretation appropriate?

Yes

Are all the source data underlying the results available to ensure full reproducibility?

Yes

Are the conclusions drawn adequately supported by the results?

Yes

Are sufficient details of methods and analysis provided to allow replication by others?

Yes

Reviewer Expertise:

high-speed data compression

I confirm that I have read this submission and believe that I have an appropriate level of expertise to confirm that it is of an acceptable scientific standard.

# Reviewer report: Reviewer response for version 2

**DOI:** 10.21956/openreseurope.25401.r80665

**Published:** 2026-09-11

**Authors:** Alexandra Theodoropoulou

**Affiliations:** 1 University of Patras, Patras, Greece

My suggestions have been sufficiently addressed. I therefore recommend the manuscript's indexation.

Is the study design appropriate and does the work have academic merit?

Yes

Is the work clearly and accurately presented and does it cite the current literature?

Partly

If applicable, is the statistical analysis and its interpretation appropriate?

Not applicable

Are all the source data underlying the results available to ensure full reproducibility?

Partly

Are the conclusions drawn adequately supported by the results?

Partly

Are sufficient details of methods and analysis provided to allow replication by others?

Partly

Reviewer Expertise:

ICT in Management, Digital Marketing, Benchmarking big-data systems and distributed ML, Decision-support analytics, Performance evaluation of data pipelines, Reproducible data engineering, Clustering for systems decisions

I confirm that I have read this submission and believe that I have an appropriate level of expertise to confirm that it is of an acceptable scientific standard.

# Reviewer report: Reviewer response for version 1

**DOI:** 10.21956/openreseurope.20832.r61752

**Published:** 2025-10-11

**Authors:** Martin Burtscher

**Affiliations:** 1 Texas State University, San Marcos, Texas, USA

Summary:

 This paper describes and evaluates COMPASS, a compression approach for databases. It clusters the columns of the database and then finds the best compression algorithm from a list of a few compressors for each cluster (i.e., group of similar columns). It significantly outperforms prior approaches that only use one or two compression algorithms for the entire database.

 Recommendations:

 The report does not include any speed results. If the operation of COMPASS is too slow, it may not be practical even if it delivers superior compression ratios. Hence, I recommend performance results be included.

 Using only a single database likely biases the results and makes them non-general. It would be good to evaluate COMPASS on a set of different databases.

 The report mentions Zstandard but does not compare to it. I have found that Zstandard works very well in practice and recommend using it as one of the compressors that can be chosen by COMPASS.

 The statement "The hypothesis of COMPASS is that [a] multiple data compression scheme can be more effective for complex big data because it allows for incremental compression and partial decompression" should be rephrased. Incremental compression and partial decompression are independent and can also be supported by a single-compression scheme.

 Citations:

 There is quite a bit of related work on compressing heterogeneous datasets with different compressors.

 [Reference 1]

 [Reference 2]

 [Reference 3]

Is the study design appropriate and does the work have academic merit?

Yes

Is the work clearly and accurately presented and does it cite the current literature?

Partly

If applicable, is the statistical analysis and its interpretation appropriate?

Yes

Are all the source data underlying the results available to ensure full reproducibility?

Yes

Are the conclusions drawn adequately supported by the results?

Yes

Are sufficient details of methods and analysis provided to allow replication by others?

Yes

Reviewer Expertise:

high-speed data compression

I confirm that I have read this submission and believe that I have an appropriate level of expertise to confirm that it is of an acceptable scientific standard, however I have significant reservations, as outlined above.

## References

[1] : Automatic synthesis of compression techniques for heterogeneous files. *Software: Practice and Experience* .1995;25(10) : 10.1002/spe.43802510031097-1116 10.1002/spe.4380251003

[2] : Evolution of human-competitive lossless compression algorithms with GP-zip2. *Genetic Programming and Evolvable Machines* .2011;12(4) : 10.1007/s10710-011-9133-6335-364 10.1007/s10710-011-9133-6

[3] : Using Machine Learning to Predict Effective Compression Algorithms for Heterogeneous Datasets.2024;

# Response

**Published:** 2026-03-18

**Authors:** Constantinos Costa

**Affiliations:** Rinnoco Ltd, Limassol, Cyprus

We would like to sincerely thank Dr. Burtscher for the constructive feedback and helpful suggestions. We have revised the manuscript accordingly, and all modifications are reflected in the new version (V2) of the manuscript.

 - Runtime evaluation

 The reviewer noted that the original manuscript did not report runtime results. Response:

 We agree that runtime performance is an important practical consideration. The revised manuscript now includes runtime measurements for compression and decompression for both COMPASS and the baseline methods. These results are presented in Table 1, allowing readers to better assess the computational overhead associated with clustering and the selection of a compression scheme. - Dataset diversity

 The reviewer pointed out that evaluating the method on a single dataset may limit generalizability. Response:

 We acknowledge that evaluating the method on a single dataset may limit generalizability. A paragraph discussing this limitation and outlining future evaluation on additional datasets has been added to the Conclusion section. - Zstandard comparison

 The reviewer recommended including Zstandard as a reference compressor. Response:

 We appreciate this suggestion. The current prototype implementation of COMPASS selects compression algorithms from those available in the Python zipfile library for simplicity and portability. We have clarified this design choice in Section 2 and noted that the framework is extensible and can incorporate additional compressors such as Zstandard or LZ4 in future implementations. - Hypothesis statement clarification

 The reviewer suggested rephrasing the hypothesis statement. Response:

 We have revised the hypothesis statement in Section 3 to clarify that the main objective of COMPASS is to exploit heterogeneity in relational datasets by selecting compression schemes tailored to groups of columns with similar statistical characteristics. Related work

 The reviewer suggested additional relevant references. Response:

 We thank the reviewer for highlighting these studies. The Related Work section has been expanded to include the suggested references on automated and heterogeneous compression techniques. We thank Dr. Burtscher again for the helpful comments and for pointing out relevant related work that strengthened the manuscript.

# Reviewer report: Reviewer response for version 1

**DOI:** 10.21956/openreseurope.20832.r61751

**Published:** 2025-10-08

**Authors:** Alexandra Theodoropoulou

**Affiliations:** 1 University of Patras, Patras, Greece

Summary of the article:

 The manuscript proposes COMPASS, a “multiple compression” approach for relational data. It partitions a table’s columns into groups and selects a compression scheme per group. Two variants are evaluated: COMPASS-D (K-Means on data values) and COMPASS-E (K-Means on column entropy). The method chooses the number of clusters using the silhouette coefficient and then “exhaustively” tests compression schemes for low-silhouette clusterings. Experiments on a real environmental monitoring database (Envmon, ~1 GB) show disk-space reductions: COMPASS variants store data at roughly 2–18.2% of RAW, versus 4–23% for baselines; best-case gains over baseline reach ~56%. Code and data are reported as available on GitHub/Zenodo.

 ----------------------------------------------------------------------

 Is the work clearly and accurately presented and does it cite the current literature?

 Partly — the main idea is clear and the citations are generally relevant, but there are wording/typo issues and a few gaps in positioning against common codecs.

 Is the study design appropriate and does the work have academic merit?

 Yes — the problem is meaningful, the approach is reasonable, and the contribution is of interest; the evaluation set could be broader, but the design itself has merit.

 Are sufficient details of methods and analysis provided to allow replication by others?

 Partly — data/code are provided, yet some key details are missing (e.g., seeds, clustering settings, small ablations), which makes exact replication harder.

 If applicable, is the statistical analysis and its interpretation appropriate?

 Not applicable — the paper is primarily systems/engineering with clustering and measurements; it does not hinge on formal statistical inference.

 Are all the source data underlying the results available to ensure full reproducibility?

 Partly — availability is claimed, but there are minor inconsistencies (e.g., DOI formatting) that should be fixed to ensure smooth access.

 Are the conclusions drawn adequately supported by the results?

 Partly — storage gains are shown convincingly; however, efficiency (time/overhead) and generalization claims would benefit from a bit more evidence or careful scoping.

 ------------------------------------------------------------------------

 SUGGESTED REVISIONS

Runtime and resources:

 It would strengthen the paper to include a few basic efficiency metrics. Even a compact table (or a short note per experiment) with compression time, decompression time, and rough CPU/memory usage would help readers interpret the practical trade-offs. If full profiling feels heavy, indicative averages with a line or two on the test environment may be enough.

Baselines and codecs

 Since many readers will be familiar with Zstd, LZ4, or Snappy, adding at least one of these as a reference point could situate COMPASS in everyday practice. If those codecs are out of scope for a reason (e.g., tooling constraints or focus on a specific library), a brief explanation would also work. Either way, a small note here would make the comparisons feel more complete.

Clustering rationale

 The choice to prefer lower silhouette values is interesting, but it may not be clear to all readers. A brief note explaining the idea—why lower values can help compression in your case—would help. If possible, add a small example table with k, average silhouette, and the resulting size/time (or a short ablation). It can be very short; just enough to show the pattern clearly.

Variability / stability

 It would help to show that results are stable. Could you run each experiment a few times (e.g., with different random seeds) and report the average plus a small error bar or standard deviation? Even 3 runs are enough. This adds credibility, especially because clustering can vary slightly.

Query-time/read-time

 Could you add 2–3 very small timing tests to show how fast data can be read after compression? For example: (a) time to read a whole table, (b) time to run a simple filter (e.g., value > threshold), and (c) time to compute an average on one column. Please report the time (in seconds) for COMPASS and for the baseline. If this is outside the paper’s scope at this time, a one-line note is also okay (e.g., “Query-time tests are valuable but out of scope; we plan them for future work”).

Study limitations / threats to validity

 It would help to add a short, dedicated limitations section. You might briefly note the dataset scope and domain, any sensitivity to the encoding choices, how the silhouette heuristic behaves across settings, and the computational overhead. If you have already checked some of these, a concise summary would be great.

Future research directions

 The conclusion could sketch a few next steps in a little more detail. For example, you might point to richer clustering features, possible learning-based selection of codecs, links to DBMS metadata/optimizers, broader codec/encoding evaluations, to name a few. Feel free to frame these in whatever way best fits your vision of the project.

Figure quality

 Figure 3 has quite low quality. It would benefit the paper if the authors could increase its quality.

Consistency and references

 To improve readability and accuracy, please correct minor errors. There are a few typographical slips—e.g., “COMPASS-E” appears as “COMAPASS-E,” and “Envmon dataset” in the Reference section is misspelled. Also, the Shannon citation is labeled as reference 31 in the text but appears as 32 in the reference list; aligning the in-text citation with the bibliography numbering would resolve this mismatch.

Is the study design appropriate and does the work have academic merit?

Yes

Is the work clearly and accurately presented and does it cite the current literature?

Partly

If applicable, is the statistical analysis and its interpretation appropriate?

Not applicable

Are all the source data underlying the results available to ensure full reproducibility?

Partly

Are the conclusions drawn adequately supported by the results?

Partly

Are sufficient details of methods and analysis provided to allow replication by others?

Partly

Reviewer Expertise:

ICT in Management, Digital Marketing, Benchmarking big-data systems and distributed ML, Decision-support analytics, Performance evaluation of data pipelines, Reproducible data engineering, Clustering for systems decisions

I confirm that I have read this submission and believe that I have an appropriate level of expertise to confirm that it is of an acceptable scientific standard, however I have significant reservations, as outlined above.

# Response

**Published:** 2026-03-18

**Authors:** Constantinos Costa

**Affiliations:** Rinnoco Ltd, Limassol, Cyprus

We would like to sincerely thank Mrs. Theodoropoulou for the constructive feedback and helpful suggestions. We have revised the manuscript accordingly, and all modifications are reflected in the new version (V2) of the manuscript. - Runtime and resource metrics

 The reviewer suggested including efficiency metrics such as compression and decompression time, as well as information about the computational environment. Response:

 We agree that runtime information is valuable for evaluating the practical implications of the proposed approach. We have therefore added runtime measurements for compression and decompression for COMPASS and the baseline methods. These results are presented in Table 1, along with a brief description of the experimental environment in Section 4.5. - Baselines and commonly used codecs

 The reviewer suggested including commonly used compression schemes such as Zstandard, LZ4, or Snappy to better situate COMPASS in practical contexts. Response:

 We appreciate this suggestion. This prototype solution implementation of COMPASS selects compression algorithms from those available in the Python zipfile library (e.g., DEFLATE, BZIP2, and LZMA). We have clarified this design choice in the implementation section and added an explanation in Section 2 that the COMPASS framework itself is not limited to these compression schemes and can be extended to incorporate additional compressors such as Zstandard or LZ4. - Clustering rationale and silhouette interpretation

 The reviewer asked for clarification regarding the use of lower silhouette values in the clustering process. Response:

 We have expanded the explanation in Section 3.2 (Selection of best performing compression) to clarify this design choice. Unlike typical clustering tasks that aim to maximize cluster separation, our objective is to group columns that exhibit similar compression behavior. In this context, lower silhouette values may produce groupings that allow compression algorithms to better exploit structural similarities across columns. - Variability and experimental stability

 The reviewer suggested reporting multiple runs with different random seeds. Response:

 To improve reproducibility, we have clarified that the clustering experiments use a fixed random seed (seed = 42). This information has been added to the methodology description in Section 4.1. - Query-time/read-time considerations

 The reviewer suggested including small experiments showing read/query performance. Response:

 We appreciate this suggestion. The focus of the current work is on compression effectiveness and storage reduction. Query-time performance is an important aspect that we plan to explore in future work. We have added a brief clarification in Section 5 of the manuscript to acknowledge this point. - Limitations and threats to validity

 The reviewer recommended including a limitations section. Response:

 We thank the reviewer for the suggestion. We have added a paragraph in the Conclusion section discussing the study's limitations, including the dataset's scope used in the evaluation, and directions for future work. - Future work

 The reviewer suggested expanding the discussion of future research directions. Response:

 The conclusion (Section 5) has been extended to include several future research directions, including richer clustering features and evaluation on additional datasets and compression schemes. - Minor corrections

 The reviewer identified several typographical and formatting issues. Response:

 We have carefully revised the manuscript to correct typographical errors (e.g., “COMAPASS-E” → “COMPASS-E”), corrected the spelling of the Envmon dataset reference, aligned the Shannon citation numbering, and improved the quality of Figure 3. We thank Mrs. Theodoropoulou again for the valuable feedback, which helped improve the clarity and completeness of the manuscript.
